# Cooperative Chloride Hydrogel Electrolytes Enabling Ultralow-Temperature Aqueous Zinc Ion Batteries by the Hofmeister Effect

**DOI:** 10.1007/s40820-022-00836-2

**Published:** 2022-04-08

**Authors:** Changyuan Yan, Yangyang Wang, Xianyu Deng, Yonghang Xu

**Affiliations:** 1 Shenzhen Key Laboratory of Advanced Materials, School of Materials Science and Engineering, Harbin Institute of Technology, Shenzhen, 518055 People’s Republic of China; 2 School of Materials Science and Hydrogen Energy, Foshan University, Foshan, 528000 China

**Keywords:** Chloride hydrogel, Electrochemical window, Cooperative effect, Hydrogen-bond, Ultralow temperature, Aqueous zinc ion battery

## Abstract

Aqueous zinc ion batteries have high potential applicability for energy storage due to their reliable safety, environmental friendliness, and low cost. However, the freezing of aqueous electrolytes limits the normal operation of batteries at low temperatures. Herein, a series of high-performance and low-cost chloride hydrogel electrolytes with high concentrations and low freezing points are developed. The electrochemical windows of the chloride hydrogel electrolytes are enlarged by > 1 V under cryogenic conditions due to the obvious evolution of hydrogen bonds, which highly facilitates the operation of electrolytes at ultralow temperatures, as evidenced by the low-temperature Raman spectroscopy and linear scanning voltammetry. Based on the Hofmeister effect, the hydrogen-bond network of the cooperative chloride hydrogel electrolyte comprising 3 M ZnCl_2_ and 6 M LiCl can be strongly interrupted, thus exhibiting a sufficient ionic conductivity of 1.14 mS cm^−1^ and a low activation energy of 0.21 eV at −50 °C. This superior electrolyte endows a polyaniline/Zn battery with a remarkable discharge specific capacity of 96.5 mAh g^−1^ at −50 °C, while the capacity retention remains ~ 100% after 2000 cycles. These results will broaden the basic understanding of chloride hydrogel electrolytes and provide new insights into the development of ultralow-temperature aqueous batteries.

**Supplementary Information:**

The online version contains supplementary material available at 10.1007/s40820-022-00836-2.

## Highlights

- The electrochemical windows of the chloride hydrogel electrolytes are enlarged by >1 V under cryogenic conditions.
- The cells based on electrolytes with high Cl^−^ concentrations can work in more severe environments.
- Based on the Hofmeister effect, the hydrogen-bond network of the electrolytes can be strongly interrupted by the addition of Li^+^.
- The full cell with the optimized hydrogel electrolyte presents impressive electrochemical performance at –50 °C.

**Supplementary Information:**

The online version contains supplementary material available at 10.1007/s40820-022-00836-2.

## Graphical abstract

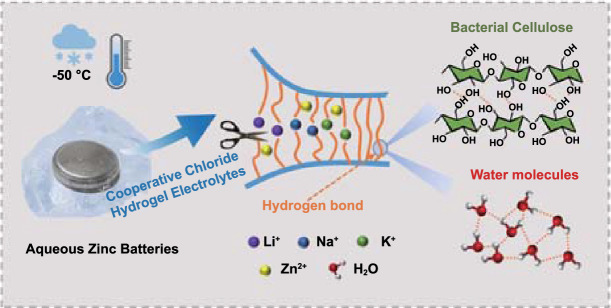

## Introduction

The rapid development of the electronic (E) era has put forward more stringent requirements for energy storage devices [1, 2]. Aqueous batteries that employ water as the electrolyte solvent fundamentally solve the intrinsic challenges caused by flammable organic electrolytes, such as safety concerns, strict manufacturing conditions and expensive electrolyte costs [3, 4]. Moreover, aqueous electrolytes serve as fast ion conductors, thereby improving the power characteristics of battery systems; thus, their use has attracted considerable attention worldwide [5].

**Fig. 1:**
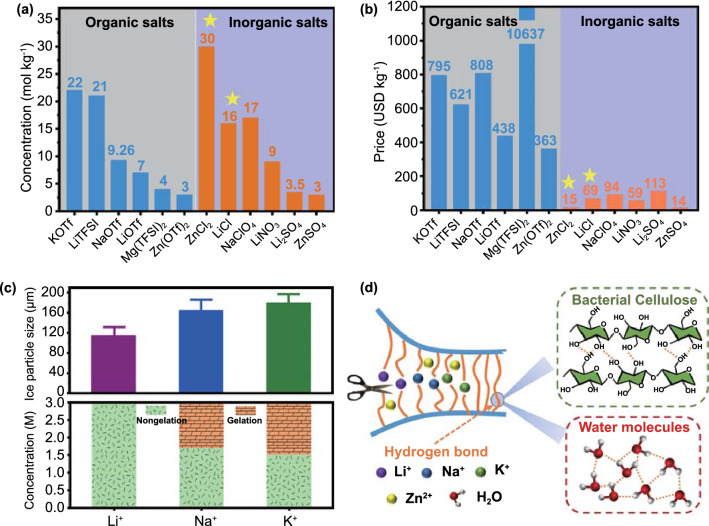
Comparison of **a** concentration and **b** price between organic salts (KOTf [50], LiTFSI [16], NaOTf [51], LiOTf [52], Mg(TFSI)_2_ [53], and Zn(OTf)_2_ [54]) and inorganic salts (ZnCl_2_ [55], LiCl [42], NaClO_4_ [56], LiNO_3_ [42], Li_2_SO_4_ [42], and ZnSO_4_ [57]). **c** Influence of Li^+^, Na^+^, and K^+^ on the size of ice crystals and crystalline state (nongelation and gelation) of PVA chains (Cl^−^ was used as the common anion) [26, 27]. **d** Schematic diagram of interrupted H-bonds between cellulose and cellulose chains, water and water molecules induced by cations (Li^+^, Na^+^, and K^+^)

Unfortunately, compared with low-melting organic electrolytes such as ethyl acetate [6], 1,3-dioxolane [7], and methyl propionate [8], aqueous electrolytes often suffer from congelation at ultralow temperatures due to the inherent freezing point (0 °C) of water molecules; this results in a decrease in battery capacity and limits the number of operating cycles of electronic devices [9, 10]. High-concentration aqueous electrolytes have recently been proposed, which have lower freezing point temperatures by preventing the formation of a hydrogen-bond (H-bond) network between the water molecules [11–14]. These aqueous electrolytes can inhibit the thermodynamic reactivity of water by adjusting the number of water molecules in the ionic solvation sheath, which further overcomes the parasitic interfacial reactions and delivers high low-temperature resistance [15]. In addition, high-concentration aqueous electrolytes play a decisive role in widening the electrochemical window and achieving an ultralong cycle life [16–18]. As shown in Fig. 1a, the concentrations of the KOTf (KCF_3_SO_3_) and LiTFSI (LiN(CF_3_SO_2_)_2_) organic salts and the ZnCl_2_, LiCl, and NaClO_4_ inorganic salts all exceed 10 m (molality, mol kg^−1^). However, the prices of the above inorganic and organic salts are quite different (Fig. 1b). For instance, the price of LiCl is only $69 per kg, which is approximately 1/9 that of LiTFSI, while ZnCl_2_ is only $15 per kg, which is approximately 1/40 that of LiTFSI. Therefore, from a cost perspective, it would be highly beneficial to develop highly concentrated aqueous electrolytes utilizing the water-soluble chloride salts ZnCl_2_ and LiCl.

In terms of aqueous zinc ion batteries, cooperative hydrated ion electrolytes have shown impressive low-temperature electrochemical performances in previous reports. For instance, a 2 M (molality, mol L^−1^) ZnSO_4_ + 4 M LiCl hydrogel electrolyte with high zinc reversibility enables the battery to achieve an ~ 100% capacity retention at −20 °C [19]. However, ZnSO_4_ is not preferred in cold climates of < −20 °C due to its low solubility at lower temperatures. It is worth noting that a highly cooperative aqueous chloride electrolyte (7.6 M ZnCl_2_ + 0.05 M SnCl_2_) successfully completed low-temperature operation for 200 cycles at −50 °C, during which zinc dendrite growth was suppressed [20]. Additionally, gel electrolytes have attracted considerable attention for their competitive ion conductivity, good flexibility, low interfacial impedance, and high integration with electrodes; thus, they have been regarded as potential replacements for liquid and all-solid-state electrolytes [21–25]. In the typical Hofmeister series, different types of cations will affect the size of ice particles after recrystallization and the crystalline state (nongelation and gelation) of poly(vinyl alcohol) (PVA) hydrogels, which led to various degrees of damage to the H-bond structure [26, 27]. As shown in Fig. 1c, the H-bond destruction in ice crystals and PVA chains by different cations but with the same Cl^−^ anion follows the order Li^+^ > Na^+^ > K^+^. The bacterial cellulose (BC) hydrogel, containing a large amount of hydroxyl groups and water molecules, is similar to the PVA hydrogel. Thus, the use of BC hydrogel as the electrolyte matrix should produce an analogous order of destruction of H-bonds by Li^+^, Na^+^, and K^+^, as shown in Fig. 1d. In principle, compared to that in the amorphous region, ion transport is much slower in the crystalline regions of BC and the ice crystal regions (where H-bonds exist), which means that fast ion transport is more likely to occur in the ion-interrupted regions of the ice crystals and the amorphous part of BC at low temperatures [28, 29]. For example, Hu et al. found that the amorphous regions of cellulose nanofibrils opened by Cu^2+^ were capable of quickly transporting ions, resulting in the high ionic conductivity and a high transference number [30]. The increase in the viscosity of electrolytes at low temperatures will lead to a sharp drop in ionic conductivity [31, 32], which may be compensated by the way the ions open more channels in the crystalline regions of the gel electrolyte. Therefore, it is rational to hypothesize that the cooperative ZnCl_2_-LiCl, ZnCl_2_-NaCl, and ZnCl_2_-KCl BC hydrogel electrolytes will exhibit unique low-temperature electrochemical performance in aqueous zinc ion batteries, and this stirred our interest. Furthermore, chlorides have been reported to potentially result in unstable electrolyte/electrode interfaces [33, 34], thereby causing the narrow electrochemical windows. However, this instability has not yet been thoroughly investigated.

Herein, we systematically studied the electrochemical window stability of BC hydrogel electrolytes composed of chlorides and other salts, and focused on the low-temperature electrochemical behaviour of highly concentrated and cooperative ZnCl_2_-LiCl, ZnCl_2_-NaCl, and ZnCl_2_-KCl hydrogel electrolytes. The low-temperature Raman spectroscopy and linear scanning voltammetry (LSV) results indicate that chloride hydrogel electrolytes (CHEs) with stainless steel as the working electrode have widened electrochemical windows of > 1 V at low temperatures because of the significant increases in the H-bond ratios of the electrolytes. The widened electrochemical windows promote the ultralow-temperature application of aqueous electrolytes in zinc batteries. Compared with other cooperative systems, 3 M ZnCl_2_ + 6 M LiCl shows stronger H-bond destruction in the hydrogel electrolyte, exposing more amorphous regions and lowering the viscosity; thus, a high ionic conductivity (*σ*) of 1.14 mS cm^−1^ and a low activation energy (*E*_*a*_) of 0.21 eV at −50 °C are obtained. This phenomenon is consistent with the Hofmeister effect. Integrating the expanded electrochemical window and the fast reaction kinetics of the cooperative chloride electrolytes, a polyaniline (PANI)/Zn battery system delivers a superior capacity of 96.5 mAh g^−1^ and a stable cycle life of 2000 cycles at −50 °C.

## Experimental Section

### Materials

ZnCl_2_, LiCl, NaCl, KCl, KF, ZnBr_2_, KI, LiN(CF_3_SO_2_)_2_ (LiTFSI), Zn(CF_3_SO_3_)_2_ (Zn(OTf)_2_), Li_2_SO_4_, ZnSO_4_, aniline, acetylene black, and polyvinylidene fluoride were purchased from Sigma-Aldrich. Ammonium persulfate (APS) was purchased from Macklin. The bacterial cellulose (BC) hydrogel was purchased from Hainan Yi De Food Co., Ltd. Carbon cloth was purchased from the Cetech Co., Ltd. LiFePO_4_ was purchased from the Shenzhen Kejing Group. Zinc foil (20 μm thick) was purchased from Qingyuan Metal Material Co., Ltd.

### Preparation of the Hydrogel Electrolytes

The frozen-thawed BC hydrogels were prepared according to our previous report [35]. The hydrogel electrolytes were prepared by immersing the frozen-thawed BC hydrogels in aqueous solutions of different salts for 24 h to reach the final equilibrium state.

### Materials Characterization

The freezing point of hydrogel electrolytes was measured by differential scanning calorimetry (DSC) through a METTLER TOLEDO DSC3 from −60 to 20 °C. The Raman spectroscopic studies were recorded at the 532 nm excitation wavelength with a confocal microscope (LabRAM HR Evolution). FTIR spectroscopy were collected using a Nicolet iS50 spectrometer. The ^1^H NMR experiments were carried out on an AVANCE NEO 600 M. XPS was performed on a spectrometer (ESCALAB Xi^+^) with Al Kα source. The surface morphologies were identified using field emission scanning electron microscopy (FESEM, HITACHI S-4700). The electrolytes’ crystalline patterns were collected on a PANalytical, Aereis X-ray diffractometer using Cu Kα radiation at room temperature. Tensile curves were acquired with an electronic universal testing machine (MDTC-EQ-M12-01) with a stretching rate of 10 mm/min.

### Electrochemical Tests

PANI was obtained by oxidizing aniline monomer with ammonium persulfate in hydrochloric acid aqueous solution followed by its in situ polymerization on carbon cloth [36]. The mass loading of PANI was about 1 mg cm^−2^. For the LiFePO_4_ cathode, in a typical process, the active material was mixed with acetylene black and polyvinylidene fluoride at an 8:1:1 weight ratio. The mass loading of the above active material was approximately 1–2 mg cm^−2^. Cells were assembled in an air atmosphere using Zn foil as the anode in CR2032-type coin cells. Galvanostatic cycling studies were conducted using a Neware battery charge/discharge system (China). Cyclic voltammetry (CV) and electrochemical impedance spectroscopy (EIS) were performed by employing an electrochemical workstation (Wuhan CorrTest, CS310H). The CV curves were measured at a scan rate of 2 mV s^−1^ from 0.6 to 1.6 V. EIS spectra were recorded with an electrochemical workstation in the temperature range of −50–25 °C and a frequency range of 0.01 to 10^6^ Hz. The electrochemical window of the hydrogel electrolytes at different temperatures was investigated with a three-electrode configuration, where stainless steel (SS) foil, Ti foil, Ni foil, Al foil, Cu foil, Pt sheet and carbon cloth (CC) were used as working electrodes and the Zn foil was used as the reference and counter electrode.

### Calculation Details

The hydrogel electrolytes were sandwiched between two stainless steel sheets, and the ionic conductivity (*σ*) at different temperatures is calculated by Eq. 1:

1 $$\sigma = \frac{d}{R \times S}$$

where *d, R,* and *S* are the thickness, the bulk resistance, and the contact area with electrode, respectively.

The cation transfer numbers (*t*^+^) are calculated by Eq. 2:

2 $$t^{ + } = \frac{{I_{s} \left( {\Delta V - I_{0} R_{0} } \right)}}{{I_{0} \left( {\Delta V - I_{s} R_{s} } \right)}}$$

where *I*_*s*_ and *I*_*0*_ are the steady-state and initial currents, respectively, and *R*_*s*_ and *R*_*0*_ are the corresponding steady-state and initial resistances, and ∆*V* is the potential applied across the symmetric Zn/Zn cell.

The activation energy (*E*_*a*_) of the hydrogel electrolytes is estimated by the fitting of Arrhenius equation (Eq. 3):

3 $$\sigma \left( T \right) = Ae^{{\left( { - \frac{Ea}{{RT}}} \right)}}$$

where *R* is the molar gas constant, *A* is a constant, and *T* is the absolute temperature.

The crystallinity of BC hydrogel electrolytes is obtained by Eq. 4:

4 $$Xc = \frac{{I_{002} - I_{am} }}{{I_{002} }} \times 100\%$$

where *I*_002_ is the maximum intensity at about 22.5°, and *I*_am_ is amorphous scattering intensity at 18.0°.

## Results and Discussion

### Electrochemical Window Study

**Fig. 2:**
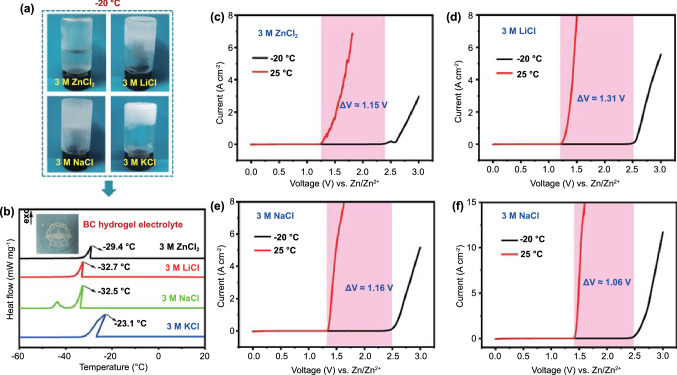
**a** Optical photographs of four chloride aqueous solutions at -20 °C. **b** DSC curves of the corresponding hydrogel electrolytes. The inset is the optical image of 3 M ZnCl_2_ hydrogel electrolyte. The electrochemical windows of hydrogel electrolytes containing **c** 3 M ZnCl_2_, **d** 3 M LiCl, **e** 3 M NaCl, and **f** 3 M KCl at 25 °C and −20 °C

We adopted frozen-thawed BC hydrogel as the electrolyte matrix (Fig. S1a). The translucent nature indicated their porous structure, which provided channels for the fast transport of ions (Fig. S1b). Moreover, the BC hydrogel had excellent crystallinity (73.26%) and was thin (Fig. S1c–d), which helped to suppress the growth of zinc dendrites and improved the battery energy density. Four chloride salts (ZnCl_2_, LiCl, NaCl, and KCl) were chosen to prepare aqueous solutions at a concentration of 3 M (Fig. 2a). Among them, the 3 M ZnCl_2_ aqueous solution did not freeze at −20 °C, while the other three chloride aqueous solutions all froze at −20 °C. Notably, the large number of hydroxyl groups in BC would break the inherent H-bonds between water molecules, resulting the BC hydrogel having a freezing point of −11.3 °C [35]. Therefore, the corresponding CHEs all had a solid–liquid transition temperature below −20 °C (Fig. 2b) [37]. For the above hydrogel electrolyte systems, the electrochemical windows at 25 and −20 °C were determined by the three-electrode configuration (stainless steel sheet as the working electrode and Zn foil as the reference electrode and counter electrode) in Fig. S1, and the test results are shown in Fig. 2c–f. At 25 °C, the electrochemical windows of the CHEs were all approximately 1.25 V. Interestingly, the electrochemical windows of the 3 M ZnCl_2_, 3 M LiCl, 3 M NaCl_2_, and 3 M KCl hydrogel electrolytes were greatly widened by 1.15, 1.31, 1.16, and 1.06 V, respectively, at −20 °C. Similar observations were attained for dilute CHEs, regardless of that whether the enlargement of the electrochemical windows at −20 °C was slightly smaller (1.09 V for 1 M ZnCl_2_, 0.96 V for 1 M LiCl, 0.96 V for 1 M NaCl, and 0.98 V for the 1 M KCl hydrogel electrolyte) (Fig. S2). In contrast, the influence of temperature on the electrochemical windows of non-CHEs (Zn(OTf)_2_, ZnSO_4_, LiTFSI, and Li_2_SO_4_) was almost negligible (Fig. S3). In addition, the electrochemical windows of the hydrogel electrolytes with other halogens (F^−^, Br^−^, and I^−^) as anions were also inspected (Fig. S4). It was clear that the electrolytes with F^−^, Br^−^, and I^−^ all exhibited a small electrochemical window expansion at −20 °C, illustrating that the enlargement of the electrochemical windows by > 1 V at −20 °C was a unique property of Cl^−^. The above results demonstrated that a low-temperature environment facilitated widening the electrochemical windows of CHEs with stainless steel as the working electrode, thereby promoting the feasibility of using the CHEs in low-temperature button batteries.

As a control experiment, the electrochemical windows of 3 M ZnCl_2_ and 3 M LiCl hydrogel electrolytes with other working electrodes (Al foil, Cu foil, Ni foil, Pt sheet, carbon cloth (CC) and Ti foil) were tested. Figure S5 and Table S1 indicate that the electrochemical windows of CHEs with non-stainless steel working electrodes are all hardly sensitive to temperature changes. Furthermore, the three-electrode configuration based on the stainless steel working electrode was tested in narrower temperature ranges by storing the electrolytes in an ice-water mixture (0 °C), refrigerator (5 °C) and freezer (-5 °C) (Fig. S6a). Figure S6b shows that the 3 M ZnCl_2_, 3 M LiCl, 3 M NaCl, and 3 M KCl hydrogel electrolytes all have a widened electrochemical window with a critical point of 0 °C. Since 0 °C is the freezing point of water molecules, the widening of the electrochemical windows of the CHEs at low temperatures should be closely related to the changes in the structure of the hydrogels, which was caused by Cl^−^.

### Evolution of H-bonds at Low Temperatures

**Fig. 3:**
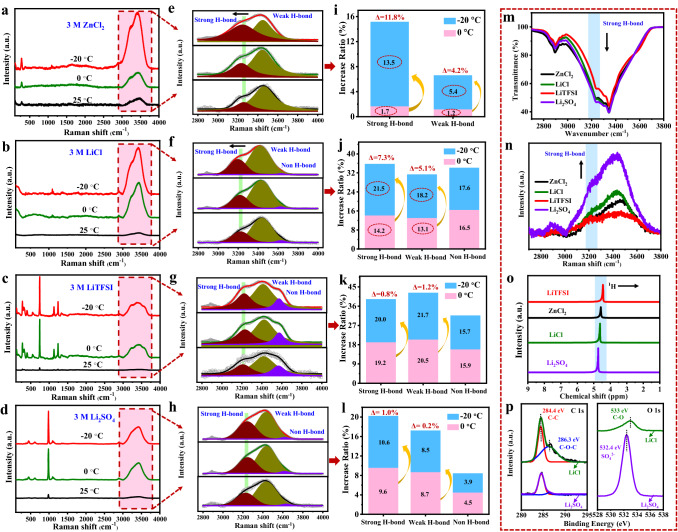
**a-d** Raman spectra of different hydrogel electrolytes (the scan range is 100–4000 cm^−1^). **e**–**h** Fitted O–H stretching vibrations represent electrolytes with strong, weak, and non-H-bonds. **i**–**l** Increase ratios of H-bonds at 0 °C and −20 °C compared with 25 °C. **m** FTIR spectra, **n** Raman spectra, and **o** ^1^H NMR spectra of different hydrogel electrolytes. **p** high-resolution C 1*s* and O 1*s* of XPS survey spectra from 3 M LiCl and 3 M Li_2_SO_4_ electrolytes

Water molecules in ice crystals are completely connected by H-bonds [38, 39]. The large number of hydrated ions generated by the addition of salts will break part of the H-bond network between water molecules, which is also the reason for lowering the freezing point of the hydrogel electrolytes [14, 35]. Hence, the behaviour of the CHEs at low temperatures were intimately associated with the H-bond structure. To deeply investigate the mechanism of widening the electrochemical windows of the CHEs at low temperatures, the H-bond structures in the CHEs (3 M ZnCl_2_ and 3 M LiCl) and non-CHEs (3 M LiTFSI and 3 M Li_2_SO_4_) at various temperatures were determined by Raman spectroscopy (Fig. 3a–d). Compared with that at 25 °C, the characteristic peaks of electrolytes were strengthened at 0 and −20 °C, which should be attributed to the stronger interaction between ions and water. Additionally, it was observed that the electrolytes did not show a new characteristic peak with decreasing temperature. Figure 3e–h clearly exhibits fitted peaks at 2800–4000 cm^−1^, which are the typical O–H stretching vibration bands of hydrogel electrolytes, and the peaks located at ~ 3240, ~ 3410, and ~ 3580 cm^−1^ correspond to strong, weak and non-H-bonds, respectively [11, 19]. For the 3 M ZnCl_2_ hydrogel electrolyte, the H-bond network was mainly composed of strong and weak H-bonds, while strong, weak, and non-H-bonds were observed with the 3 M LiCl, 3 M LiTFSI, and 3 M Li_2_SO_4_ counterparts. In addition, compared with the non-CHEs, the stretching vibration peaks of the strong H-bonds in the CHEs showed a notable redshift (to a lower wavenumber) as the temperature gradually decreased, implying increased H-bond interaction [40].

To further quantify the influence of temperature on the H-bonds, the increase ratios of H-bonds were calculated by integrating the fitting peak areas (Fig. 3i–l). It is worth mentioning that when the temperature was lowered from 0 to −20 °C, the increase ratio of the strong H-bonds in the 3 M ZnCl_2_ hydrogel electrolyte increased from 1.7% to 13.5% (Δ = 11.8%), and that of the 3 M LiCl electrolyte increased from 14.2% to 21.5% (Δ = 7.3%). In contrast, for the 3 M LiTFSI and 3 M Li_2_SO_4_ hydrogel electrolytes, the increase ratios of the strong H-bonds at 0 and −20 °C were similar (Δ = 0.8% and 1.0%, respectively). Raman spectroscopy confirmed that lower temperatures promoted the formation of H-bonds in the CHEs, which agreed with the LSV curves. Thus, the expanded electrochemical windows of the CHEs at low temperatures were believed to be due to the formation of more H-bonds, which could be explained by the stronger inhibition of the water activity kinetics. Moreover, the H-bond strength in different anionic electrolytes (Cl^−^, SO_4_^2−^, and TFSI^−^) was further examined through FTIR, Raman, and ^1^H NMR spectroscopies to reveal whether there was a close connection between chlorine and H-bonds. As shown in Fig. 3m, n, the intensity of the strong H-bond peak located at 3240 cm^−1^ followed the order SO_4_^2−^ > Cl^−^ > TFSI^−^, and this was consistent with the sequence in which the ^1^H chemical shifts to lower field (the weaker disruption of H-bond structure) in Fig. 3o. Above results indicated that Cl^−^ has a stronger ability to break H-bond than SO_4_^2−^, which was due to the fact that the electronegative O^2−^ in SO_4_^2−^ are more likely to form H-bonds with OH^−^ of BC hydrogel (Fig. S8a). Figures 3p and S8b-c further exhibited the XPS survey spectra of 3 M LiCl and 3 M Li_2_SO_4_ electrolytes. As compared with that of Li_2_SO_4_ electrolyte, the C–O–C (286.3 eV) and C–O (533 eV) of C 1*s* and O 1*s* spectra presented a higher binding energy in the LiCl electrolyte. Hence, the H-bond strength of CHEs at room temperature was weaker, while the hydration of Cl^−^ was more stable with the decrease in temperatures [41], which greatly promoted the increase in the H-bond ratio.

### Matching of a High Cl^−^ Concentration with Harsh Conditions

**Fig. 4:**
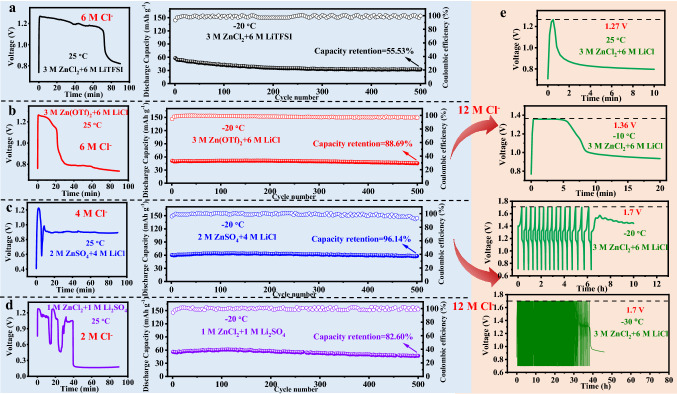
GCD tests of the LiFePO_4_/Zn cells with **a** 3 M ZnCl_2_ + 6 M LiTFSI, **b** 3 M Zn(OTf)_2_ + 6 M LiCl, **c** 2 M ZnSO_4_ + 4 M LiCl and **d** 1 M ZnCl_2_ + 1 M Li_2_SO_4_ hydrogel electrolytes at 25 °C and −20 °C, respectively (0.2 A g^−1^). **e** GCD tests of the LiFePO_4_/Zn cells with 3 M ZnCl_2_ + 6 M LiCl hydrogel electrolytes at 25, −10, −20, and −30 °C (0.2 A g^−1^)

Based on the expanded electrochemical windows of the CHEs and exceptional performance of the stainless steel working electrode at low temperatures, the cycling stability of LiFePO_4_/Zn button battery was studied (Fig. 4a–d). Li^+^ and Zn^2+^ were introduced into each electrolyte as the working mechanism of the LiFePO_4_/Zn battery converted from the single-ion transport of a “rocking chair” battery into double-ion transport. Moreover, to study the relationship between the concentration of Cl^−^ and temperature adaptability, other anions were introduced to adjust the concentration of Cl^−^. Therefore, we employed various cooperative hydrogel electrolytes, namely 3 M ZnCl_2_ + 6 M LiTFSI (6 M Cl^−^), 3 M Zn(OTf)_2_ + 6 M LiCl (6 M Cl^−^), 2 M ZnSO_4_ + 4 M LiCl (4 M Cl^−^), and 1 M ZnCl_2_ + 1 M Li_2_SO_4_ (2 M Cl^−^). Because of the ionic effect, O atoms were bound by salt ions through hydration and were absent from the formation of H-bonds, resulting in the feasible use of these hydrogel electrolytes at low temperatures. Regarding the galvanostatic charge–discharge (GCD) curves in a voltage range of 0.7–1.7 V, the as-built button batteries all exhibited a severe micro-short circuit at approximately 1.25 V (25 °C). All the LiFePO_4_/Zn batteries exhibited superior performance, lasting for 500 cycles at -20 °C, especially the battery with the 2 M ZnSO_4_ + 4 M LiCl hydrogel electrolyte, which showed the best discharge specific capacity (62.5 mAh g^−1^) and capacity retention (96.14%) at a current density of 0.2 A g^−1^. In addition, a comparison of electrolytes with the same concentrations of Zn^2+^ (2 M) and Li^+^ (4 M) was performed (Fig. S9a–c). Note that the similar micro-short circuit and cycling stability (discharge specific capacity of ~ 65 mAh g^−1^ and capacity retention of ~ 100%) of the batteries occurred at room temperature and low temperature, respectively. Meanwhile, the capacity at low temperatures is mainly limited by the LiFePO_4_ electrode.

At −20 °C (Fig. S10a), the Zn/Zn symmetric battery using 3 M ZnCl_2_ hydrogel electrolyte displayed stable Zn plating/stripping for over 210 h at 0.2 mA cm^−2^, and the overpotential was only 30 mV. However, at 25 °C, the symmetric Zn/Zn battery showed a significant increase in thickness after cycling, and the overpotential increased from 30 to 130 mV after only 4.6 h, which was accompanied by the generation of cuboid by-products on the corresponding spring (Fig. S10b-d). Additionally, compared with the morphology of the bare Zn foil, we observed that although the corresponding Zn anode had a similar metallic lustre after 7.5 h of stripping/plating cycles, there was still obvious zinc dendrite growth (Fig. S10e-f). The above results strongly demonstrated that the CHEs had better low-temperature stability. Taking into account the solubility and cost of chloride salts, the 3 M ZnCl_2_ + 6 M LiCl electrolyte with a high concentration of Cl^−^ (12 M) was employed in a LiFePO_4_/Zn button battery (Fig. 4e). The same cycling process was repeated at different temperatures. The cut-off voltages of the full battery for sudden failure at 25 and −10 °C were 1.27 and 1.36 V, respectively. Note that the corresponding battery did not immediately fail to work at −20 and −30 °C but stopped working after 7 and 38 h of the charging/discharging process, respectively. Therefore, batteries based on electrolytes with high Cl^−^ concentrations were capable of working in more severe environments (such as those in electric vehicles, aerospace, and military applications), including operations at lower temperatures and higher current densities.

### Reaction Kinetics at Low Temperatures Based on the Hofmeister Effect

**Fig. 5:**
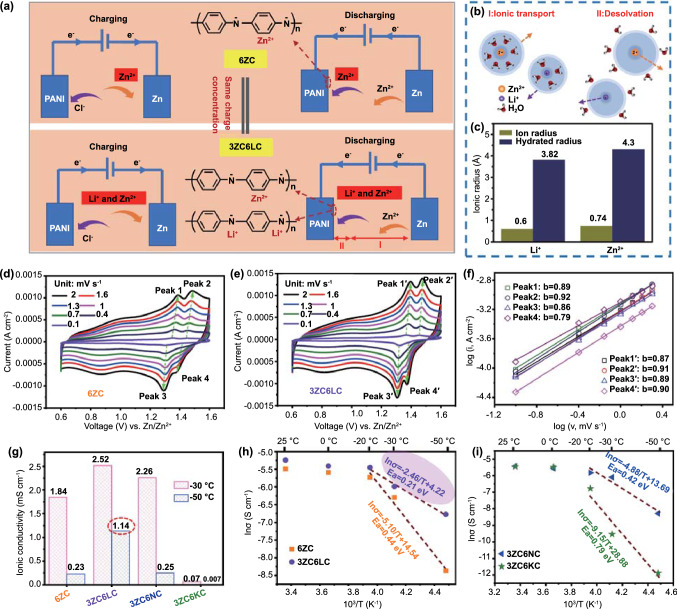
**a** Redox mechanism of PANI/Zn batteries with 6ZC and 3ZC6LC, respectively. **b** Schematic diagram of the transport and desolvation of hydrated zinc ion and lithium ion. **c** The ionic radius and hydrate ion radius of zinc ion and lithium ion. CV curves of full PANI/Zn batteries using **d** 6ZC and **e** 3ZC6LC at various scan rates from 0.1 to 2.0 mV s^−1^ (−30 °C). **f** Corresponding determination of the b values. **g** The *σ* of hydrogels electrolytes. **h** Arrhenius plots of 6ZCand 3ZC6LC, respectively. **i** Arrhenius plots of 3ZC6NC and 3ZC6KC

Inorganic materials generally store energy through ion insertion/extraction. However, lower temperatures will greatly reduce the diffusion coefficient of ions in the electrode materials or even hinder ion insertion, leading to rapid capacity decay. For example, the battery assembled with a commercial LiCoO_2_ cathode only maintained approximately 10% of its room-temperature capacity when operating at < −30 °C [42]. In contrast, the charge storage of organic cathode materials is mainly by the coordination of ions and redox active groups on molecular chains, resulting in faster reaction kinetics; thus, it is easier to obtain a higher capacity at low temperatures [43]. The electrochemical performance of the 6 M ZnCl_2_ and 3 M ZnCl_2_ + 6 M LiCl hydrogel electrolytes (denoted as 6ZC and 3ZC6LC, respectively), both of which had the same charge concentration of anions/cations, were studied in PANI/Zn button cells at ultralow temperatures. Compared with pure CC (Fig. S11a), the PANI distributed on the surface of CC was dark green, featuring a stamen-like nanostructure (Fig. S11b). The ion storage mechanism of the PANI/Zn battery is shown in Fig. 5a. During the discharge process, the Zn^2+^/Li^+^ cations interacted with the negatively charged C–N^−^ of the reduced PANI, and during the charging process, the Cl^−^ anions interacted with the positively charged C–N^+^ of the oxidized PANI [36]. The cyclic voltammetry (CV) curves of PANI/Zn cells were obtained at rates from 0.1 to 2.0 mV s^−1^ (Fig. 5d, e). The two pairs of redox peaks in the CV curves of batteries with 6ZC and 3ZC6LC remained the same. The current responses at various sweep rates are analysed by Eq. 5 [44, 45]:

5 $$i = av^{b}$$

For a semi-infinite diffusion-controlled redox process, the peak current *i* varies with *v*^0.5^ (*b* = 0.5); in contrast for a surface-controlled capacitive process, it varies with *v* (*b* = 1.0). Figure 5f shows the relationship between log (*i*) and log (*v*). The *b* values obtained by the slopes of the fitted curves were approximately 1.0, implying that the redox reaction kinetics were mainly dominated by surface-controlled ion intercalation behaviour. The detailed capacitive contributions (the magenta area) at 1 mV s^−1^ are shown in Fig. S12. Thus, the impact of ion transport and charge transfer (ion desolvation) in the electrolytes on the reaction kinetics of the full battery was considered (Fig. 5b). The radii of Li^+^ and its hydrate ions were smaller than those of Zn^2+^ and its corresponding hydrate ions (Fig. 5c), which might lead to faster diffusion kinetics in the hydrogel electrolytes [46]. Figure 5g shows the σ of electrolytes at low temperatures, which were calculated based on the Nyquist curves of Fig. S13. At −30 °C, both 6ZC and 3ZC6LC achieved high σ values of 1.84 and 2.52 mS cm^−1^, respectively. Due to the drop in temperature, the σ of 6ZC decreased to 0.23 mS cm^−1^ at −50 °C, while 3ZC6LC retained a high σ of 1.14 mS cm^−1^, which was much higher than the reported aqueous electrolyte using dimethyl sulfoxide as an additive (0.11 mS cm^−1^ at −50 °C) [47]. In addition, compared to 6ZC (0.44 eV), 3ZC6LC exhibited a lower *E*_*a*_ of 0.21 eV at −50 °C, signifying stronger ion desolvation and faster interfacial charge transfer (Fig. 5h). Compared with 6ZC, because 3ZC6LC showed a higher pH value and no freezing point above −60 °C (Fig. S14), it was believed that the higher *σ* of 3ZC6LC at −50 °C was not contributed by H^+^ motion and the solid–liquid transition temperature. Furthermore, the σ values of hydrogel electrolytes with the same charge concentrations and different molar mass ratios of ZnCl_2_/LiCl at −50 °C were analysed (Fig. S15). The experimental results collectively implied that a high concentration of LiCl was beneficial for improving the σ of electrolytes at ultralow temperatures, probably due to the small ionic radius of Li^+^.

To uncover whether the ionic radius was the critical factor affecting σ at ultralow temperatures, the *σ* values of the 3 M ZnCl_2_ + 6 M NaCl and 3 M ZnCl_2_ + 6 M KCl hydrogel electrolytes (denoted 3ZC6NC and 3ZC6KC, respectively) were further evaluated. Na^+^ has the same valence state as Li^+^ in aqueous solution, and their hydrated ion radius is also close (3.82 Å for [Li(H_2_O)_4_]^+^ and 3.58 Å for [Na(H_2_O)_4_]^+^) [46]. Figure 5g, i implies that 3ZC6NC has a σ of 0.25 mS cm^−1^ and an *E*_*a*_ of 0.42 eV, which indicates that the ionic radius is not the dominant factor for σ at ultralow temperatures. Additionally, the σ of 3ZC6KC at −50 °C was 0.007 mS cm^−1^, and the corresponding *E*_*a*_ was 0.79 eV. Figure S16 and Table S2 show the cation transference number (*t*^+^) measured with symmetric Zn/Zn cell. Compared with 3ZC6NC and 3ZC6KC, higher values of approximately 0.85 (at −30 °C) and 0.79 (at −50 °C) were obtained for 3ZC6LC, which indicated that 3ZC6LC had lower ion transfer resistance at low temperatures. Notably, the results above were consistent with the sequence of H-bond destruction of water molecules and BC chains based on the Hofmeister effect, Li^+^ > Na^+^ > K^+^, collectively certifying Li^+^ as a superior cooperative cation for the electrolytes of zinc batteries. Additionally, σ could be further reflected by combining the Nernst-Einstein equation and the Stokes–Einstein equation [48, 49], and the rearranged equation is shown in Eq. 6:

6 $$\sigma = \frac{{nq^{2} }}{6\pi \eta r}$$

where *η* is the viscosity of the electrolyte, *r* is the Stokes radius of the ions, *n* is the ion concentration, and *q* is the ion charge. Equation (6) shows that the rapid kinetics of the ion are determined by the ionic radius and electrolyte viscosity in the case of the same ion charge concentration. In this regard, the ions of ZnCl_2_-LiCl effectively broke the crystalline regions of both water and cellulose (Fig. S17), thereby reducing the *η* of the electrolyte to strengthen the ion kinetics at ultralow temperatures. Moreover, Fig. S18a shows the stress–strain curves of the hydrogel electrolytes (3ZC6LC, 3ZC6NC, and 3ZC6KC) to demonstrate the destruction of the BC crystalline region by ions. As expected, the hydrogel electrolytes had similar tensile strengths (~ 13 MPa) that were lower than that of the BC hydrogel matrix (~ 15 MPa). Note that 3ZC6LC exhibits a greater elongation due to the more H-bonds being broken by ions in the hydrogel. Most pores were in the range of 50–160 nm and had analogous shapes in the hydrogel electrolytes (Fig. S18b). No significant differences in the mechanical properties and morphology were observed between the hydrogel electrolytes, implying that the H-bond disruption order of the electrolytes based on the Hofmeister effect only became more obvious at low temperatures.

### Ultralow Temperature Aqueous PANI/Zn Battery Constructed with the ZnCl_2_-LiCl Hydrogel Electrolyte

**Fig. 6:**
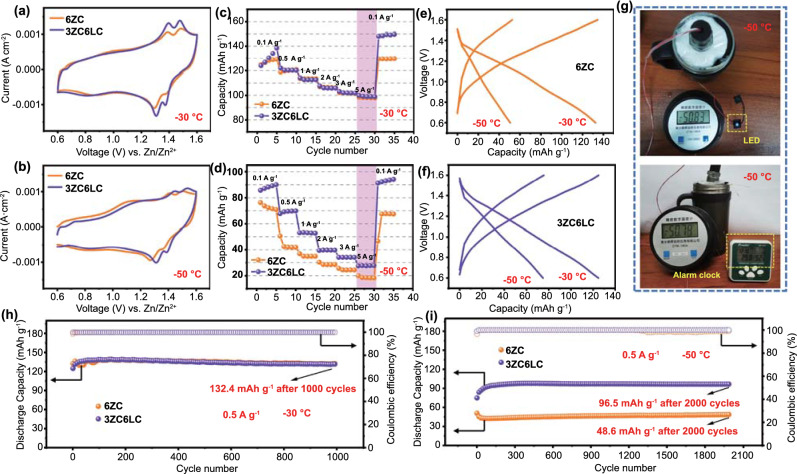
CV curves of PANI/Zn cells in various electrolytes at **a** −30 °C and **b** −50 °C (2 mV s^−1^). Rate performance of the PANI/Zn cells at **c** −30 °C and **d** −50 °C. Charge–discharge profiles (0.5 A g^−1^) of the PANI/Zn cells at **e** -30 °C and **f** -50 °C. **g** The photos of a LED and an alarm clock powered by three PANI/Zn coin cells connected in series at -50 °C. Cycling performance and coulombic efficiency of the PANI/Zn cells using 6ZCand 3ZC6LC at **h** -30 °C and **i** -50 °C

The expanded electrochemical window and excellent reaction kinetics of 3ZC6LC discussed above encouraged us to systematically evaluate the electrochemical performance of a PANI/Zn button cell. In the CV curves at −30 °C (Fig. 6a), the batteries with 3ZC6LC and 6ZC both showed favourable reversible oxidation and reduction peaks, in accordance with their outstanding rate performance and resilience capabilities (Fig. 6c). In terms of the more rigorous working condition of −50 °C, the dynamics of the full cell with 6ZC dramatically decreased (Fig. 6b), while 3ZC6LC still retained a better rate performance and specific discharge capacity (Fig. 6d). The charge–discharge curves in Fig. 6e, f further prove that 3ZC6LC has high redox reversibility and low temperature tolerance. EIS spectra were obtained to probe the charge-transfer resistance (*R*_*ct*_) of the symmetric Zn/Zn batteries and full batteries at low temperatures (Fig.S19). At −30 and −50 °C, the higher slope and smaller semicircle diameter of the EIS curves of the batteries indicated a low *R*_*ct*_ (< 10 Ω), which further demonstrated the good interfacial compatibility between 3ZC6LC and the electrode. As expected, the PANI/Zn button battery with an electrolyte having a high Cl^−^ concentration also underwent a micro-short circuit after running at −30 °C for 30 h (3ZC6LC)/120 h (6ZC) at a low current density of 0.2 A g^−1^ (Fig. S20), which was similar to the results of the LiFePO_4_/Zn battery.

To verify the previous hypothesis, cycling performance tests were conducted under more severe conditions (0.5 A g^−1^ at −30/−50 °C), which would be essential for the practical application of low-temperature batteries. Figure 6h shows the ultralong steady cycling of the battery (0.5 A g^−1^ at −30 °C), exhibiting a discharge specific capacity of 132.4 mAh g^−1^, a high capacity retention of > 95% and a coulombic efficiency of 100% after 1000 cycles. When the temperature was decreased to −50 °C, the capacity of the battery with 6ZC decreased rapidly in the first 30 cycles and remained 48.6 mAh g^−1^ after 2000 cycles. Notably, the battery based on 3ZC6LC had an obvious activation process in the initial cycles and then exhibited durable the cycling performance of a capacity of 96.5 mAh g^−1^ for over 2000 cycles (approximately 30 days) at 0.5 A g^−1^ (Fig. 6i). Even at a high mass loading of ~ 3 mg cm^−2^, the PANI/Zn battery still exhibited a high rate capability and prominent cycling stability at −30 °C (capacity of 97.9 mAh g^−1^ and capacity retention of ~ 100% after 1000 cycles), which benefitted from the excellent ion transport of 3ZC6LC (Fig. S21). From the self-discharge curves in Fig. S22a, it could be observed that the PANI/Zn battery with 6ZC had a more significant voltage drop at the initial stage of standing. After a natural month, the batteries with 6ZC and 3ZC6LC could still maintain a stable open-circuit voltage above 1.28 V at −50 °C. Moreover, three button cells in series could successfully power LEDs and alarm clocks at −50 °C (Fig. 6g). In addition, the corresponding polarization curves (Fig. S22b) also showed no obvious irreversible fluctuation in overpotential within a month of operation at an extremely low temperature of −50 °C, confirming that the electrochemical performance of this system was preserved. Hence, all the experimental results demonstrated that the cooperative hydrated ions of 3ZC6LC resulted in a promising electrolyte solution for use in high-performance PANI/Zn batteries, allowing for their normal, long-term operation at ultralow temperatures (Table S2).

## Conclusion

The low-temperature electrochemical behaviour of a series of CHEs was systematically probed in this work. The experimental analysis validates the mechanism that electrochemical windows of the CHEs widen by > 1 V, namely there are significant increases in the H-bond ratios at lower temperatures. Based on the Hofmeister effect, Li^+^ has a stronger ability to break the H-bonds within water molecules and cellulose chains than Na^+^ and K^+^; thus, the addition of Li^+^ results in the electrolyte having a lower viscosity at low temperatures. Thus, among the highly concentrated cooperative ZnCl_2_-LiCl, ZnCl_2_-NaCl, and ZnCl_2_-KCl systems, the cooperative 3 M ZnCl_2_ + 6 M LiCl hydrogel electrolyte achieves the most sufficient *σ* of 1.14 mS cm^−1^ and lowest *E*_*a*_ of 0.21 eV at −50 °C. Benefiting from the expanded electrochemical window and superior reaction kinetics, an assembled PANI/Zn button battery exhibits an impressive discharge capacity of 96.5 mAh g^−1^ at 0.5 A g^−1^ and ultralong cycling stability with a high-capacity retention of ~ 100% after 2000 cycles at −50 °C. This work provides new ideas and pathways for designing high-performance, low-temperature aqueous batteries by using cost-effective cooperative chloride hydrogels as electrolytes.

## Supplementary Information

Below is the link to the electronic supplementary material.

Supplementary file1 (DOCX 19303 KB)
