# Supplementary material for: Cooperative Chloride Hydrogel Electrolytes Enabling Ultralow-Temperature Aqueous Zinc Ion Batteries by the Hofmeister Effect
Source: Nanomicro Lett. 2022 Apr 8;14:98. doi: 10.1007/s40820-022-00836-2 (PMC8993986; doi:10.1007/s40820-022-00836-2)
Supplement: Supplementary file 1 — Supplementary file1 (DOCX 19303 KB) [file 40820_2022_836_MOESM1_ESM.docx]

Supporting Information for

**Cooperative Chloride Hydrogel Electrolytes Enabling Ultralow-Temperature Aqueous Zinc Ion Batteries** **by the Hofmeister Effect**

Changyuan Yan^1^, Yangyang Wang^1^, Xianyu Deng^1,^ *, Yonghang Xu^2,^ *

^1^Shenzhen Key Laboratory of Advanced Materials, School of Materials Science and Engineering, Harbin Institute of Technology, Shenzhen, 518055, China

^2^School of Materials Science and Hydrogen Energy, Foshan University, Foshan, 528000, China

*Corresponding authors. E-mail: [xydeng@hit.edu.cn](mailto:xydeng@hit.edu.cn) (Xianyu Deng); [yonghangxu@fosu.edu.cn](mailto:yonghangxu@fosu.edu.cn) (Yonghang Xu)

**Supplementary Figures**

**Fig. S1** Optical image (**a**), SEM image (**b**), XRD pattern (**c**) and cross-sectional SEM image (**d**) of the BC hydrogel

**Fig. S2** The three-electrodes configuration for measuring the electrochemical windows of the electrolytes

**Fig. S3** The electrochemical windows of hydrogel electrolytes containing 1 M ZnCl_2_, 1 M LiCl, 1 M NaCl and 1 M KCl at 25 °C and -20 °C, respectively

**Fig. S4** The electrochemical windows of hydrogel electrolytes containing 3 M Zn(OTf)_2_, 3 M ZnSO_4_, 3 M LiTFSI and 3 M Li_2_SO_4_ at 25 °C and -20 °C, respectively

**Fig. S5** The electrochemical windows of hydrogel electrolytes containing 1 M KF, 1 M ZnBr_2_, 1 M KI at 25 °C and -20 °C, respectively

**Fig. S6** The electrochemical windows of 3 M ZnCl_2_ and 3 M LiCl hydrogel electrolytes based on different working electrodes at 25 °C and -20 °C, respectively

**Fig. S7** (**a**) The temperature control devices (5 °C, 0 °C and -5 °C). (**b**) The electrochemical windows of hydrogel electrolytes containing 3 M ZnCl_2_, 3 M LiCl, 3 M NaCl and 3 M KCl at 5 °C, 0 °C and -5 °C, respectively

__

**Fig. S8** (**a**) Schematic diagram of the H-bond formation between SO_4_^2-^ and bacterial cellulose/water molecules. (**b**) XPS spectra for 3 M LiCl hydrogel electrolyte (Li 1s and Cl 2p). (**c**) XPS spectra for 3 M Li_2_SO_4_ hydrogel electrolyte (Li 1s and S 2p)

__

**Fig. S9** GCD tests of the LiFePO_4_/Zn cells using hydrogel electrolytes with 2 M Zn^2+^ and 4 M Li^+^ (2 M ZnCl_2_+4 M LiTFSI (**a**), 2 M Zn(OTf)_2_+4 M LiCl (**b**), and 2 M ZnCl_2_+2 M Li_2_SO_4_ (**c**)) at 25 °C and -20 °C, respectively (0.2 A g^-1^)

__

**Fig. S10** The voltage profiles of symmetric Zn/Zn cell with 3 M ZnCl_2_ hydrogel electrolyte at -20 °C (**a**) and 25 °C (**b**), respectively (0.2 mA cm^-2^). Optical photographs (**c**), SEM images of the spring (**d**) and Zn anode (**e**) from the corresponding Zn/Zn cell after cycling at 25 °C. (**f**) SEM image of bare Zn foil (the inset is the optical image)

**Fig. S11** SEM images of carbon cloth (**a**) and PANI/carbon cloth (**b**). Insets are the corresponding optical images

**Fig. S12** Capacitive-controlled and diffusion-controlled contributions of PANI/Zn batteries based on 6ZC (**a**) and 3ZC6LC (**b**) at a scan rate of 1 mV s^−1^ (-30 ℃)

**Fig. S13** EIS spectra of the symmetric SS/SS system based on 6ZC (**a**), 3ZC6LC (**b**), 3ZC6NC (**c**) and 3ZC6KC (**d**) measured at different temperatures

**Fig. S14** The pH values (**a**) and DSC curves (**b**) of 6ZC and 3ZC6LC

**Fig. S15** (**a**) EIS spectra of the symmetric SS/SS system based on 1 M ZnCl_2_+10 M LiCl, 2 M ZnCl_2_+8 M LiCl, 4 M ZnCl_2_+4 M LiCl and 5 M ZnCl_2_+2 M LiCl hydrogel electrolytes at -50 °C (denoted 1ZC10LC, 2ZC8LC, 4ZC4LC and 5ZC2LC, respectively). (**b**) The ionic conductivity of corresponding hydrogel electrolytes

**Fig. S16** Current-time curves at -30 °C (**a-c**) and -50 °C (**d-f**) of symmetric Zn/Zn cells following a voltage of 10 mV for 1000 s. Insets are the corresponding EIS spectra before and after polarization

**Fig. S17** XRD patterns of 3ZC6LC, 3ZC6NC and 3ZC6KC

**Fig. S18** (**a**) Stress–strain curves of BC, 3ZC6LC, 3ZC6NC and 3ZC6KC. (**b**) SEM images of corresponding hydrogel electrolytes after immersing in deionized water for 12 h

**Fig. S19** EIS spectra of Zn/Zn (**a**) and PANI/Zn (**b**) cells with 3ZC6LC before testing

**Fig. S20** The voltage profiles of PANI/Zn cells with 6ZC and 3ZC6LC at -30 °C and 0.2 A g^-1^

**Fig. S21** Rate performance (**a**) and cycling performance (**b**) of the PANI/Zn cell with high mass loading (~3 mg cm^-2^) by using 3ZC6LC at -30 °C

**Fig. S22** (**a**) The self-discharge curves of PANI/Zn cell with 6ZC and 3ZC6LC at -50 °C (30 days). (**b**) The voltage profiles of symmetric Zn/Zn cell with 6ZC and 3ZC6LC at -50 °C and 0.2 mA cm^-2^ (30 days). (**c**) The temperature control equipment (-50 °C)

**Table S1** The electrochemical windows of 3 M ZnCl_2_ and 3 M LiCl hydrogel electrolytes at different working electrodes

| **Working electrodes** | **3 M ZnCl_2_** | | **3 M LiCl** | |
| --- | --- | --- | --- | --- |
|  | **25 °C** | **-20 °C** | **25 °C** | **-20 °C** |
| **stainless steel** | **1.26 V** | **2.39 V** | **1.20 V** | **2.51 V** |
| **Al** | 0.27 V | 0.28 V | 0.29 V | 0.37 V |
| **Cu** | 0.73 V | 0.75 V | 0.84 V | 0.85 V |
| **Ni** | 0.76 V | 0.84 V | 0.98 V | 1.09 V |
| **Pt** | 2.02 V | 2.04 V | 2.09 V | 2.14 V |
| **CC** | 2.41 V | 2.16 V | 2.26 V | 2.29 V |
| **Ti** | 2.22 V | 2.32 V | 2.32 V | 2.46 V |

**Table S2** Calculation of transference numbers from analysis of polarization experiments at low temperatures

| **Electrolyte** | **Temperature(°C)** | **ΔV(mV)** | **R_0_(Ω)** | **Rs (Ω)** | **I_0_(μA)** | **Is(μA)** | **t^+^** |
| --- | --- | --- | --- | --- | --- | --- | --- |
| **3ZC6LC** | **-30** | **10** | **1.06** | **1.08** | **7.28** | **6.18** | **0.85** |
| **3ZC6NC** | -30 | 10 | 0.87 | 2.91 | 7.19 | 6.32 | 0.88 |
| **3ZC6KC** | -30 | 10 | 45.09 | 47.22 | 2.27 | 1.51 | 0.66 |
| **3ZC6LC** | **-50** | **10** | **2.09** | **3.06** | **3.72** | **2.94** | **0.79** |
| **3ZC6NC** | -50 | 10 | 14.36 | 17.74 | 1.92 | 1.27 | 0.66 |
| **3ZC6KC** | -50 | 10 | 13967 | 14911 | 0.12 | 0.07 | 0.52 |

**Table S3** Comparison of low-temperature performance of PANI/Zn batteries with other electrolytes reported in the literatures

| **Cathode**  **material** | **Electrolyte** | **Operating**  **temperature** | **Cycle life** | **Capacity** | **Ref.** |
| --- | --- | --- | --- | --- | --- |
| PANI | 2 M ZnSO_4_+ 50 v/v% methanol | -10 °C | 89.3% after 2000 cycles at 1 A g^-1^ | 100.8 mAh g^-1^ | [S1] |
| PANI | 1 M ZnCl_2_+0.5 M NH_4_Cl+50 wt% EG | -20 °C | 99.8% after 150 cycles at 1 A g^-1^ | 73.9 mAh g^–1^ | [S2] |
| PANI | 7.5 m ZnCl_2_ | -70 °C | 100% after 2000 cycles at 0.2 A g^-1^ | 84.9 mAh g^-1^ | [S3] |
| **PANI** | **3 M ZnCl_2_+6 M LiCl** | **-50 °C** | **100% after 2000 cycles at 0.5 A g^-1^** | **96.5 mAh g^-1^** | **This work** |

**Supplementary References**

[S1] Q. Zhang, K. Xia, Y. Ma, Y. Lu, L. Li, J. Liang, S. Chou, J. Chen, Chaotropic anion and fast-kinetics cathode enabling low-temperature aqueous Zn batteries, ACS Energy Lett. **6**(8) (2021) 2704-2712. <https://doi.org/10.1021/acsenergylett.1c01054>

[S2] Z. Cong, W. Guo, P. Zhang, W. Sha, Z. Guo, C. Chang, F. Xu, X. Gang, W. Hu, X. Pu, Wearable antifreezing fiber-shaped Zn/PANI batteries with suppressed Zn dendrites and operation in sweat electrolytes, ACS Appl. Mater. Interfaces **13**(15) (2021) 17608-17617. <https://doi.org/10.1021/acsami.1c02065>

[S3] Q. Zhang, Y. Ma, Y. Lu, L. Li, F. Wan, K. Zhang, J. Chen, Modulating electrolyte structure for ultralow temperature aqueous zinc batteries, Nat. Conmun. **11**(1) (2020) 4463. <https://doi.org/10.1038/s41467-020-18284-0>
